# The Effect of Different Opioids on Acid-Base Balance and Blood Gas Analysis in Hospitalized Dogs

**DOI:** 10.3389/fvets.2022.802186

**Published:** 2022-03-17

**Authors:** Fausto Quintavalla, Kevin Pascal Spindler, Raffaella Aldigeri, Francesca Fidanzio

**Affiliations:** ^1^Department of Veterinary Sciences, University of Parma, Parma, Italy; ^2^Department of Medicine and Surgery, University of Parma, Parma, Italy

**Keywords:** dog, glucose, lactate, electrolytes, tramadol, methadone, buprenorphine

## Abstract

Pain management is central to veterinary practice, contributing to successful case outcomes and enhancement of the veterinarian-client-patient relationship. Analgesic drugs represent one of the pillars of the multimodal approach to acute and chronic pain management. In dogs, the most used opioids are methadone, buprenorphine and tramadol. Several episodes of hypoglycemia in people treated with tramadol and methadone have recently been described. The aim of this work is to evaluate the changes in the glycemic and acid-base balance induced by tramadol, methadone and buprenorphine in hospitalized dogs. A retrospective review of the medical records of dogs hospitalized for both medical and surgical reasons was performed. During 2018-2020, a total of 876 canine patients were treated with opioids, including 228 with tramadol, 273 with methadone and 375 with buprenorphine. Of all these dogs, only a small percentage met the inclusion criteria presented in the initial design. All the hospitalized animals were monitored daily through clinical examination and blood sampling. Blood samples were obtained before opioid administration (T0), and 24 h (T1) and 48 h (T2) after °pioid administration. The following parameters were evaluated: blood gas value (pH, pCO_2_), acid-base state (cHCO_3_), oxymetric values (ctHb, haematocrit), electrolyte values (K+, Na+, iCa, Cl-) and metabolic values (glucose, lactate, anion GAP K+c). The glycemic value in enrolled dogs showed a decrease over time, regardless of the type of opioid used, but remained within the physiological range. The highest average glycemic drop was recorded for methadone, between T0 and T1, followed by tramadol between T1 and T2, while buprenorphine recorded the highest overall glycemic drop between T0-T2 when compared to the other two opioids. Female dogs showed the greatest drop in glycemic value. Lactate concentration always presented values beyond the physiological range at an early stage, which then normalized quickly. Measurement of electrolyte concentrations showed a consistent increase in the values of iCa, Na and Cl. In hospitalized dogs treated with opioids monitoring of gas analytic parameters is important and more attention should be paid to patients hospitalized with certain metabolic and endocrine diseases.

## Introduction

Pain management is central to veterinary practice, contributing to successful case outcomes and enhancement of the veterinarian-client-patient relationship, and various guidelines for dogs and cats have been developed in recent years (1, 2). Pain management in hospitalized dogs is a major concern and influences recovery time, quality of life, and surgical outcome (3). In particular, postoperative pain produces undesirable effect such as loss of appetite, self-trauma, maladaptive physiological responses or maladaptive behaviors, which prolong the recovery time (4).

Analgesic drugs represent one of the pillars of the multimodal approach to acute and chronic pain management, and postoperative pain assessment involves the use of opioids. Opioids are drugs that have opiate-like activities and are usually divided into four groups: full agonist, agonist-antagonist, partial agonist, and antagonists (1).

In dogs, the opioids most often used are methadone, buprenorphine and tramadol (3, 5). These opioids are metabolized in the liver through various enzyme pathways and even buprenorphine is deemed to be an effective agent for detoxification from opioids (6). Methadone is a potent synthetic opiate drug belonging to the group of full agonists. It has agonist affinity for both the μ and ∂ opioid receptors, it acts as an inhibitor at the presynaptic N-methyl-D-aspartate (NMDA) receptors, and it blocks the reuptake of noradrenaline and serotonine in the periaqueductal gray matter (7). Buprenorphine is a semi-synthetic opioid, partial agonist of μ opioid receptors, and is also a potent antagonist of *k* receptors.

Both are highly lipophilic and highly bound to plasma proteins, predominantly a glycoprotein, that is an acute reactive protein fluctuating according to several clinical conditions and concomitant drugs (8). Among the atypical centrally-acting opioids is tramadol hydrochloride, a synthetic 4-phenyl-piperidine analog of codeine but with reduced respiratory effects. Tramadol is an agonist for the μ receptors, μ opioid receptors and noradrenergic and serotoninergic systems receptors that increase serotonin release and inhibit norepinephrine reuptake (9, 10).

Opioids can produce reversible behavioral and physiological side effects in dogs (11). Patients receiving pain management must be monitored via re-examination and laboratory testing at prescribed intervals to assess for efficacy and adverse events (2). It is well known that prolonged use of opioids in human and laboratory animals may result in adverse consequences, including significant metabolic, neuroendocrine and immune effects (12, 13). Not all opioids have similar effects. Methadone did not affect the tested immune parameters, tramadol enhances NK cell activity, lymphocyte proliferation, and IL-2 release compared to morphine, while buprenorphine doesn't show any effects on the immune response compared to morphine (14). In dogs, opioid-mediated adverse effects include restlessness, dizziness headache, unsteady gait, reduced spontaneous activity, hypotension, miosis, salivation, vomiting, constipation, urinary retention, itchiness, dry mouth (15). As consequence of this, clinical parameters and pain scales, in association serum values (for instance, glucose), may provide important information about different pain conditions and proposed treatments in dogs (16).

Recently, several episodes of hypoglycemia in people treated with tramadol (17–26) and methadone (5, 27–32) have been described.

The aim of this work is to evaluate the changes in the glycemic and acid-base balance induced by tramadol, methadone and buprenorphine in hospitalized dogs.

## Materials and Methods

### Design

A retrospective review of the medical records of dogs, hospitalized for both medical and surgical reasons, at Veterinary University Hospital (VUH) of Dept. of Veterinary Medical Sciences - University of Parma between January 2018 and December 2020 was performed.

The study protocol was approved by the Ethics Committee for Animal Experimentation (PROT.94 N.14/CESA /2021).

Inclusion criteria for the study were: being hospitalized for at least 3 days; undergoing monotherapy with one of the following opioids: tramadol, methadone or buprenorphine; receiving analytical blood gas examination at admission, before opioid administration (T0), and 24 h (T1) and 48 h after opioid administration (T2) and receiving the same food during the hospitalization (Intestinal Exclusion® – Dorado srl, Monsole di Cona, Venice - Italy). Surgical patients must have received the same anesthesiological protocol consisting of premedication with midazolam (Midazolam, B.Braun, Melsungen, Germany) + dexmedetomidine (Dexdomitor®, Orion Corporation, Finland), induction with propofol (Proposure®, Boehringer Ingelheim Animal Health Italy, Milan - Italy) and maintenance with isofluorane 2%. Intraoperative analgesia must have been provided with fentanyl (Fentadon®, Dechra Veterinary Products, Turin - Italy).

Exclusion criteria were as follows: diseases that could interfere with blood glucose (diabetes mellitus, hyperadrenocorticism, pheochromocytoma, epilepsy, pancreatitis, acute hepatitis, neoplasms, systemic inflammatory response syndrome and sepsis), diester in the bitch, receiving corticosteroids or glucose infusions during hospitalization and previous opioid treatment within the past 30 days. Information recorded included signalment, body weight, medical therapy prior hospitalization, clinical presentation, physical examination findings, reason for hospitalization and details of any previous surgeries.

A control group (no analgesic treatment) was not included in this study because it is considered unethical.

### Data Collection

All the hospitalized animals were monitored daily through clinical examination and blood sampling. Blood sampling was carried out from the jugular vein in animals fasted for 8 hours between 07:00 and 8:00 a.m. The blood sample was placed in a tube containing lithium heparin and blood gas analysis (ABL 800 Flex Radiometer, Denmark) was performed within 10 minutes for the evaluation of the following parameters:

- Blood gas values: pH [7.350–7.450], pCO2 [33.6–41.2 mmHg].
- Acid-base state: cHCO3 [20.8–24.2 mmol/L].
- Oxymetric values: hemoglobin [ctHb 13.9–19.0 mg/dL], haematocrit [Hct 39.0–54.0%].
- Electrolyte values: potassium [K+ 4.1–5.3 mEq/L], sodium [Na+ 145–154 mEq/L], ionized calcium [iCa2+ 1.29–1.40 mmol/L], chloride [Cl- 105–116 mEq/L].
- Metabolic values: glucose [Glu 85–125 mg/dl], lactate [Lac 0.6–1.9 mmol/L], anion GAP K+, c [13–22 mmol/L].
- Hospitalized dogs were treated with fluid therapy with Ringer's lactate and opioid drugs to counter post-operative pain. They were treated with either tramadol at a dose of 3 mg/kg intravenous (IV) TID (Altadol®, Formavet srl, Milan - Italy), methadone at a dose of 0.2 mg/kg IV q 4 h (Semfortan®, Dechra Veterinary Products, Turin - Italy), or buprenorphine at a dose of 10 ug/kg IV TID (Buprenodale®, Dechra Veterinary Products, Turin - Italy).

Other medications that could be administered during the hospitalization period included: NSAIDs (meloxicam 0.1 mg/kg IV SID), gastroprotectant medications (omeprazole 1 mg/kg IV, BID, sucralphate 1g/dog PO TID), antiemetic medications (maropitant 1 mg/kg IV SID) and antibiotics (ampicillin + sulbactam 25 mg/kg IV TID and enrofloxacin 7.5 mg/kg IV SID).

The subjects were divided into groups based on the opioid administered: group 1 was treated with tramadol, group 2 was treated with methadone and group 3 was treated with buprenorphine.

### Statistical Analysis

Given the retrospective observational design of the study, the sample was not calculated a priori but determined by the cases available in the time interval considered. Continuous variables are expressed as mean ± standard deviation (SD) or median (interquartile range IQR) for skewed distributed data. Skewed data (glucose, lactate, sodium, potassium, chloride, bicarbonate) were log-transformed in order to achieve normal distribution as confirmed by the Kolmogorov-Smirnov test. Baseline comparisons of parameters between treatment groups were performed using ANOVA. Temporal change in parameters was analyzed by means of GLM for repeated measures with group, time, sex and surgery status as main factors. A *p*-value ≤ 0.05 was considered significant. Statistical analysis was carried out by using SPSS v. 27 (IBM Statistics).

## Results

**Figure 1:**
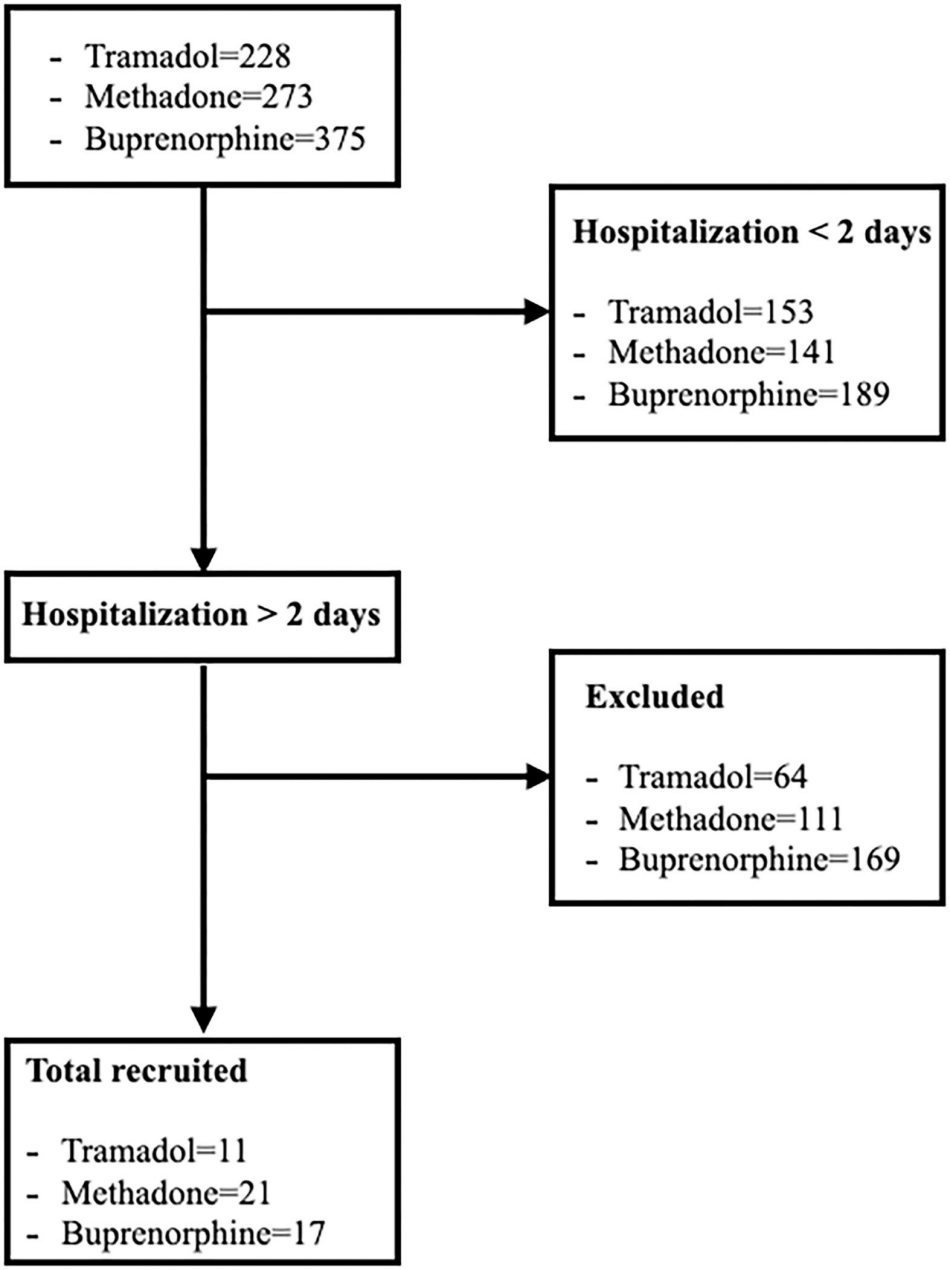
Flow diagram illustrating enrollment, inclusion, allocation process of dogs hospitalized and treated with tramadol, methadone and buprenorphine.

In the period between January 2018 and December 2020, a total of 876 patients were treated with opioids, including 228 with tramadol, 273 with methadone and 375 with buprenorphine. Of all these dogs, only a small percentage met the inclusion characteristics presented in the initial design (Figure 1 and Table 1). Thirty patients were hospitalized for surgical conditions [9 gastrointestinal foreign body, 7 pyometra, 4 gastric dilatation and volvulus (GDV), 2 haemoabdomen, 2 hit by car (HBC), 2 Wild Boar Injury, 1 intervertebral disc disease, 1 nephrolithiasis, 1 haemometra and 1 cholecystitis)] and 19 for medical reasons [13 gastroenterocolitis, 3 HBC, 1 gastrointestinal foreign body, 1 mastitis and 1 High Rise Syndrome (HRS)]. In total 11 dogs were in group 1, with an average weight of 17.54 kg consisting of 3 male and 8 female and with an average age of 7.42 years There were 21 dogs in group 2, 12 male and 9 female, with an average weight of 26.47 kg and an average age of 7.62 years. In group 3 there were 17 dogs, 10 female and 7 males, with an average weight of 23.23 kg and an average age of 6.8 years. Overall, the average age of all enlisted animals was 7.32 years with an average body weight of 23.34 kg.

**Table 1:** Dogs enrolled in Groups 1, 2 and 3 dogs.

**Opioid**	**N°** **dog**	**Breed**	**Age**	**Sex**	**Weight (kg)**	**Pathology/** **surgery**
**Tramadol**	1	Jack russell terrier	12 y 6 M	M	7.1	Gastroenterocolitis /NS
	2	Jack russell terrier	4 y 1 m	F	6.6	Intervertebral disc disease /S
	3	Caucasian shepherd dog	5 y 5 m	F	52	Pyometra /S
	4	Spanish sighthound	7 y	F	23.2	Gastroenterocolitis /NS
	5	Dogue de bordeaux	9 m	F	32.2	Gastroenterocolitis /NS
	6	CKCS^1^	7 y 4 m	F	6.7	Colecistitis /S
	7	Mongrel	4 y 1 m	M	8.1	Gastroenterocolitis /NS
	8	Mongrel	13 y 7 M	F	10.9	Pyometra /S
	9	Mongrel	2 y 2 m	M	10.4	Gastroenterocolitis /NS
	10	Mongrel	14 y 1 M	F	16.2	Pyometra /S
	11	Mongrel	10 y	F	19.5	Mastitis /NS
**Methadone**	1	Dachshund kurzhaar	1 y 8 m	M	30	HBC^2^ /NS
	2	Italian greyhound	4 y 5 m	M	14.2	Wild Boar Injury /S
	3	English bulldog	7 y 11 M	F	27	Nephrolitiasis /S
	4	German shepherd	6 y 4 m	F	28.2	Gastrointestinal foreign body /S
	5	Czechoslovakian wolfdog	9 y 1 m	F	36.7	Haemoabdomen /S
	6	Mongrel	18 y 2 M	M	7	Gastroenterocolitis /NS
	7	German shepherd	1 y 11 M	F	19.5	Gastrointestinal foreign body /S
	8	Bernese mountain dog	5 y 11 M	M	50.7	HBC^2^ /S
	9	Dobermann	9 y 11 M	M	46	GDV^3^ /S
	10	Mongrel	9 y	F	30.7	GDV^3^ /S
	11	Dobermann	2 y 9 m	F	27.5	Gastrointestinal foreign body/*S*
	12	Great dane	2 y 8 m	M	55.8	Gastroenterocolitis /NS
	13	English setter	15 y 7 M	F	17.8	Haemometra /S
	14	German pinscher	3 y 4 m	M	9	HBC^2^ /NS
	15	Mongrel	1y 6 m	M	19.6	High rise syndrome /NS
	16	Mongrel	6 y	M	21.9	Gastrointestinal foreign body /S
	17	Boxer	8 y 7 m	F	26.6	Haemoabdomen /S
	18	Mongrel	13 y 5 M	M	24	Gastroenterocolitis /NS
	19	Italian greyhound	10 y 8 M	M	39.2	GDV^3^ /S
	20	Griffon bleu de gascogne	8 y 1 m	F	20	Wild boar injury /S
	21	Maltese dog	15 y 1 M	M	4.5	HBC^2^/S
**Buprenorphine**	1	Italian mastiff	5 y 1 m	F	38	Pyometra /S
	2	Yorkshire terrier	12 y 2 M	F	4.8	Pyometra /S
	3	Mongrel	4 y 7 m	M	27.3	Gastrointestinal foreign body /S
	4	Mongrel	13 y 3 M	F	32.9	Pyometra /S
	5	Mongrel	4 y 3 m	M	14.7	Gastrointestinal foreign body /S
	6	Weimaraner	2 y 11 M	F	29	Gastroenterocolitis /NS
	7	CKCS^1^	8 y 6 m	M	10.6	Gastrointestinal foreign body /S
	8	Pug	9 y 9 m	F	7	Gastroenterocolitis /NS
	9	German shepherd	4 y 7 m	M	38	Gastroenterocolitis /NS
	10	Italian mastiff	2 y 6 m	F	52.3	Gastrointestinal foreign body /S
	11	Bernese mountain dog	8 y 7 m	M	43.9	GDV^3^ /S
	12	German shepherd	3 y 9 m	F	30.8	Gastroenterocolitis /NS
	13	Dachshund	2 y 2 m	M	4,5	Gastrointestinal foreign body /S
	14	Volpino italiano	8 y 1 m	F	10	Gastroenterocolitis /NS
	15	Gordon setter	9 y 11 M	F	20	HBC^2^ /NS
	16	CKCS^1^	9 y 9 m	F	12.7	Pyometra /S
	17	English setter	6 y 1 m	M	18.5	Gastrointestinal foreign body /NS

*M, male; F, female; y, year; m, month; S, surgery; NS, no surgery*.

1 *Cavalier King Charles spaniel;*

2 *, Hit by car;*

3 *Gastric dilatation volvolus*.

The results of the blood parameters studied for each treatment are presented as mean ± SD and median at T0, T1 and T2 in Table 2.

**Table 2:** Average, SD and median values of the various parameters analyzed at T0, T1 and T2 in Groups 1, 2 and 3 dogs.

**GROUP 1**		**pH**	**HC T** **(%)**	**pCO *2* (a)**	**HCO 3** **(b)**	**AnGa p (b)**	**AB E** **(b)**	**Na (b)**	**K** **(b)**	**Cl (b)**	**iCa** **(b)**	**La c (b)**	**Glu** **(c)**
T 0	Mean	7.3 3	49.8 5	40.5 5	20.27	13.95	−3.78	146.0 0	3.8 4	115.2 7	1.2 5	2.2 9	101.1 8
	SD	0.0 6	9.05	7.00	2.46	5.18	4.01	3.795	0.2 9	3.744	0.0 3	0.8 8	13.97
	Median	7.3 3	50.5 0	39.7 0	20.80	13.90	−3.80	145.0 0	3.9 0	115.0 0	1.2 4	2.7 0	101.0 0
T 1	Mean	7.3 0	47.7 7	44.4 6	20.55	12.27	−4.07	146.1 8	4.0 7	117.7 3	1.2 5	1.9 9	100.8 2
	SD	0,0 6	8.74	8.98	2.94	4.66	3.64	6.26	0.3 2	6.26	0.0 7	1.5 1	11.46
	Median	7.3 4	45.7 0	41.1 0	20.90	11.70	−2.90	144.0 0	4.1 0	117.0 0	1.2 5	1.5 0	100.0 0
T 2	Mean	7.3 3	44.3 0	40.9 6	21.07	10.97	−3.76	145.5 5	4.0 9	117.4 5	1.2 5	1.3 8	94.36
	SD	0,0 6	5.40	5.27	3.10	4.77	3.92	3.11	0.4 8	3.30	0.0 4	1.0 5	9.03
	Median	7.3 4	43.0 5	40.9 0	21.10	9.80	−4.20	145.0 0	4.2 0	117.0 0	1.2 7	1.0 0	96.00
**GROUP 2**		**pH**	**HC T** **(%)**	**pCO** ***2*** (a)	**HCO 3** **(b)**	**AnGa p (b)**	**AB E** **(b)**	**Na (b)**	**K** **(b)**	**Cl (b)**	**iCa** **(b)**	**La c (b)**	**Glu** **(c)**
	Mean	7.3	51.5	41.5	21.56	13.31	-	142.8	3.8	112.1	1.2	2.3	111.8
		4	0	2			3.33	6	8	0	3	8	6
T 0	SD	0.0 8	10.6 1	7.74	4.14	3.92	5.05	6.43	0.6 7	10.50	0.9 5	1.4 3	21.76
	Median	7.3 5	51.3 0	40.8 0	20.30	14.50	−4.60	145.0 0	3.8 0	115.0 0	1.2 5	2.1 0	102.0 0
T 1	Mean	7.3 5	45.5 4	40.1 3	21.94	11.26	−2.60	145.3 3	3.8 4	116.4 8	1.2 5	1.4 2	104.1 9
	SD	0,0 8	9.75	5.74	3.76	3.58	4.25	6.76	0.6 1	8.52	0.0 9	0.6 3	14.90
	Median	7.3 7	44.9 0	40.0 0	21.80	11.10	−2.90	146.0 0	3.8 0	118.0 0	1.2 6	1.3 0	100.0 0
T 2	Mean	7.3 3	43.7 0	41.7 7	21.59	11.32	−2.84	145.3 3	3.9 5	116.1 4	1.2 6	1.4 6	102.1 4
	SD	0,0 8	8.26	4.82	3.55	3.21	3.92	3.26	0.4 3	6.26	0.0 8	1.0 3	16.94
	Median	7.3 2	44.0 0	41.6 0	20.80	10.60	−2.90	145.0 0	3.8 0	116.0 0	1.2 7	1.0 0	99.00
**GROUP 3**		**pH**	**HC T** **(%)**	**pCO 2 (a)**	**HCO 3 (b)**	**AnGa p (b)**	**AB E** **(b)**	**Na (b)**	**K** **(b)**	**Cl (b)**	**iCa** **(b)**	**La c (b)**	**Glu** **(c)**
T 0	Mean	7.3 5	52.1 6	38.7 4	21.29	12.79	−3.06	143.2 4	4.9 5	113.7 1	1.2 2	1.6 5	114.7 1
	SD	0.0 6	5.90	4.53	3.42	4.04	3.96	3.88	0.7 1	5.38	0.0 6	0.9 2	25.92
	Median	7.3 6	50.6 5	40.0 0	22.20	12.60	−2.30	142.0 0	3.9 0	113.0 0	1.2 2	1.4 0	107.0 0
	Mean	7.3 3	48.0 1	40.4 0	21.06	11.78	−3.47	145.1 2	3.8 1	116.1 8	1.2 5	1.5 0	109.0 0
T 1	SD	0.0 7	8.14	7.37	3.56	4.59	4.29	3.35	0.4 2	4.97	0.0 4	0.5 2	17.45
	Median	7.3 4	46.6 0	39.0 0	21.90	11.70	−2.70	145.0 0	3.9 0	116.0 0	1.2 6	1.4 0	101.0 0
T 2	Mean	7.3 4	44.5 9	42.1 0	21.56	10.74	−2.82	147.1 2	3.9 0	118.0 6	1.2 8	1.2 6	104.2 9
	SD	0.0 5	9.93	6.46	2.72	4.02	3.59	3.74	0.3 6	4.31	0.0 6	0.3 9	12.51
	Median	7.3 4	44.1 5	41.0 0	21.50	9.90	−2.40	146.0 0	3.9 0	118.0 0	1.2 7	1.2 0	103.0 0

*(a), mmHg; (b), mmol/l; (c), mg/dl; ABE, actual base excess*.

The ANOVA analysis showed no difference at baseline (T0) in the three groups. GLM analysis for repeated data on all parameters showed a significant decrease over time for Hct (*p* < 0.0001), Anion gap (*p* < 0.0001), lactate (*p* < 0.0001) and glucose (*p* = 0.01) and a significant increase for Na (*p* = 0.01), Cl (*p* < 0.0001), iCa (*p* = 0.005) without showing a significant effect of the treatment (Table 3). The parameters were then analyzed and the effect of the surgery was also assessed. The GLM analysis for repeated data confirmed the previous results, showing a significant time increase of chlorine in the animals with surgical conditions (*p* = 0.007).

**Table 3:** GLM analysis for repeated data on all parameters.

	**Time effect**	**Group effect**	Time^*^group effect
pH	*P =* 0.51	*P =* 0.54	*P =* 0.69
HCT	*P < * 0.0001^***^	*P =* 0.89	*P =* 0.69
pCO2	*P =* 0.14	*P =* 0.72	*P =* 0.26
AnGap	*P < * 0.0001^***^	*P =* 0.91	*P =* 0.64
HCO3	*P =* 0.53	*P =* 0.59	*P =* 0.76
Base excess	*P =* 0.67	*P =* 0.78	*P =* 0.95
Sodium	*P =* 0.01^*^	*P =* 0.65	*P =* 0.09
Potassium	*P =* 0.57	*P =* 0.76	*P =* 0.25
Chloride	*P =* 0.01^*^	*P =* 0.67	*P =* 0.66
Ionized calcium	*P =* 0.005^**^	*P =* 0.98	*P =* 0.14
Lactate	*P < * 0.0001^***^	*P =* 0.31	*P =* 0.37
Glucose	*P =* 0.01^*^	*P =* 0.13	*P =* 0.91

* *p < 0.05,*

** *p < 0.01*

*** *p < 0.001*.

**Figure 2:**
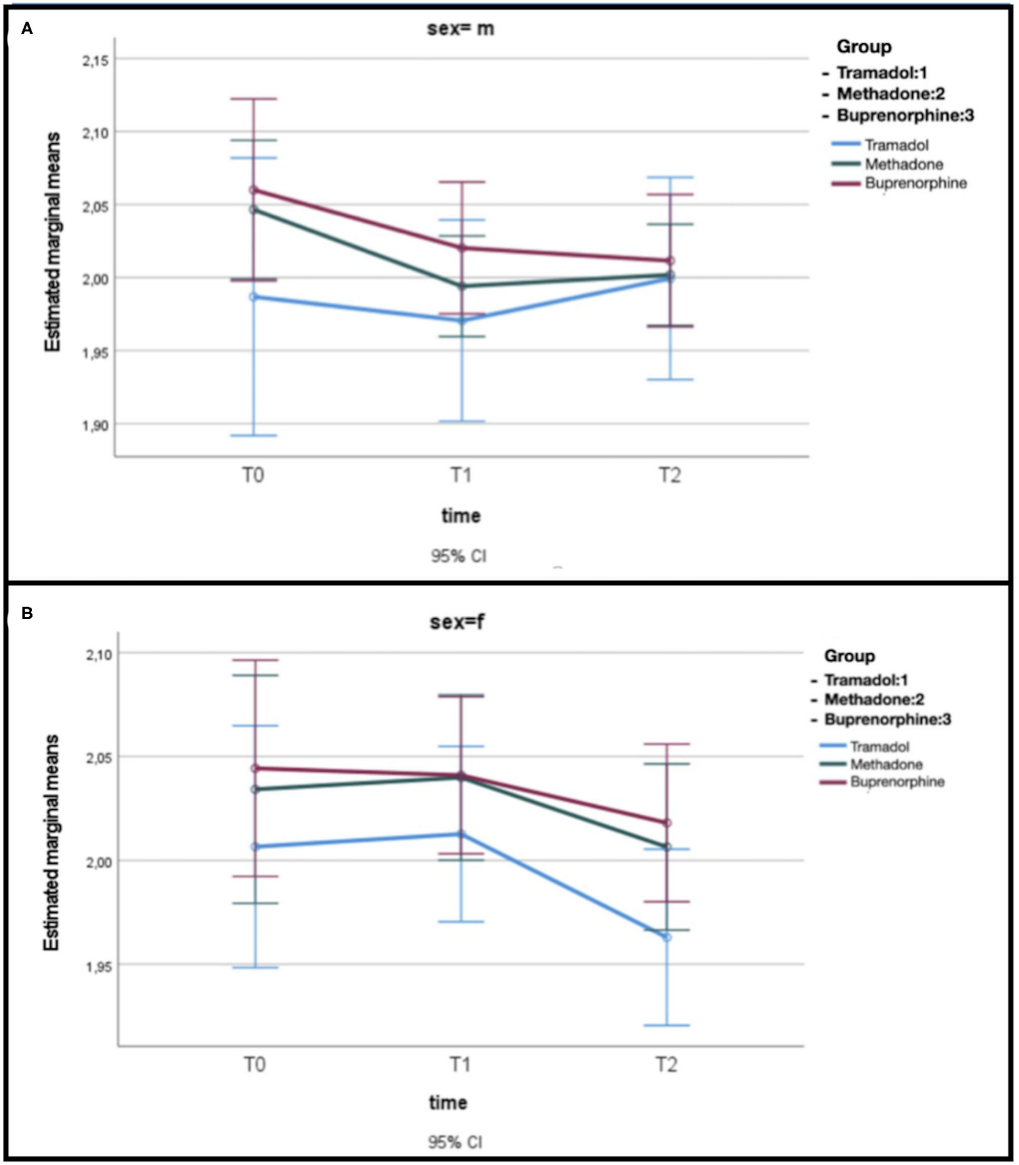
**(A)** Estimated mean glucose levels related to opioid treatment in male dogs. **(B)** Estimated mean glucose levels related to opioid treatment in female dogs.

Considering gender, the model GLM evidences a decrease in the time of glycemia (*p* = 0.03), independently from the sex and the treatment (Figure 2), where glucose is represented in log10 scale.

## Discussion

The relief of pain during the hospitalization is essential to improve the recovery and comfort of dogs. Postoperative pain shows individual variations unrelated to the duration and extent of the surgical trauma. As a stress factor, pain may be associated with increases in cortisol and glucose concentrations (16). Opioids are one of the most important drugs classes for the management of acute and chronic pain conditions and the management of postoperative pain. Besides the analgesic effects, they may interfere with the values of certain biochemical-clinical parameters, especially blood glucose and, in emergency room, the mortality is higher in dogs with hyperglycemia or hypoglycemia compared to those with normoglycemia (33, 34).

Disturbances in glucose metabolism are a known effect of pain (35) and abnormal and prolonged increased blood glucose concentrations in hospitalized human patients have been associated with brain death, fatal cardiac arrhythmia and could impact immune function and predispose patients to infection (36, 37). The effect of opiates on human blood glucose levels has been reported with contradictory results in different studies, while literature showed that medication such as NSAID's, antibiotics, propofol, and isoflurane have no or small influence on blood glucose levels (5, 38). In human medicine, reports about hypoglycemia have increased in the last 10 years and two drugs, tramadol and methadone, have been identified to be associated with higher risk (5). In our study, the glycemic value in enrolled dogs showed a decrease in its value over time, regardless of the type of opioid used but remained within the physiological range. The highest average glycemic drop was recorded for methadone between T0 and T1 (7.67 mg/dl), followed by tramadol between T1 and T2 (6.46 mg/dl), whilst buprenorphine recorded the highest overall glycemic drop between T0-T2 (10.42 mg/dl) compared to the other two opioids. Mastrocinque and Fantoni (39) used tramadol for the management of early postoperative pain following ovariohysterectomy after pyometra in dogs, they found glucose concentrations remained stable during the entire procedure. The episodes of hypoglycemia do not reappear after the suspension of the administration of the tramadol in accordance with observations by Grandvuillemin et al. (17).

Tramadol directly reduces hepatic gluconeogenesis and enhances peripheral glucose utilization in diabetic rats (40). According to Odonkor and Chhatre (21), tramadol may cause rebound hypoglycemia via interference of the intrinsic euglycemia-restoration pathways and a blunted autonomic counter-regulatory response to antecedent hypoglycemia. The mechanism by which methadone causes hypoglycemia is not well understood. Methadone at high doses clearly lowers glucose levels in a dose-dependent manner in mice (41). There is evidence for a direct action of methadone on pancreatic islets (32). According to Toce et al. (31) hyperinsulinism is the mechanism responsible for methadone-associated hypoglycemia. Methadone can also potentiate peripheral glucose utilization (42). Serotonin can lower glucose levels in mice by increasing insulin levels, and several opioids including methadone and tramadol have known serotonergic action (43). Buprenorphine, a μ receptor agonist and k receptor antagonist, apparently did not cause hypoglycemia in human patients (32).

In our study, interestingly the statistically significant difference in dogs treated with opioids appears to be based on sex. Female dogs showed the greatest drop in glycemic values; this finding was noted also by Golightly et al. (24) in hospitalized female patients.

Lactate is a biomarker of cellular energy deficit. Both in emergency and elective surgical patients, increased blood lactate levels indicate that the patient is at risk of increased morbidity and decreased changes of survival (44). Blood lactate concentration should be measured at admission for animal triage, for the identification of compensated or decompensated shock states, and during hospitalization for treatment monitoring. In dogs, blood lactate concentration has been shown to be of prognostic value in patients with gastric dilatation volvulus, severe or complicated babesiosis, septic peritonitis, and in dogs admitted to intensive care units (45, 46).

Increased blood lactate concentration was demonstrated in 3% of female dogs with pyometra (47). Overall, venous plasma lactate concentration could not discriminate between hospital survivors and non-survivors (48). The plasma lactate concentrations differed among blood samples from various sites (49), for this reason it was chosen to take blood samples only from the jugular vein. Lactate concentration in our study always presented values beyond the physiological range at an early stage which then normalized quickly (Table 2). The fall in lactate values between T0 and T2 is comparable with respect to groups treated with tramadol and methadone, while in the group treated with buprenorphine the recorded decrease was much lower.

Significant reductions in Hct and anion gap values are attributable to the underlying pathological condition and probably to the treatments carried out, rather than to the choice of administered opioid. Anion gap represents the difference in concentration between measured cations and anions in plasma. It is commonly used for assessment of the accuracy of laboratory data and analysis of acid-base disorders. According to the extensive literature review undertaken by us, there seems to be no association no between these two parameters in the dog and the choice of opioid administered.

Measurement of electrolyte concentrations is an important component of the assessment of dogs in emergency rooms or intensive care units, particularly sodium, potassium, corrected chloride and ionized calcium concentration (50). Hypochloremia and metabolic alkalosis are common in dogs with gastrointestinal foreign bodies (51, 52), and gastric dilation-volvulus syndrome (53). Our study shows a consistent increase in the values of iCa, Na and Cl. The latter shows a significant temporal increase in dogs undergoing surgical treatment. Chloride is the major anion in the extracellular fluid and is important in the metabolic regulation of acid-base balance. Serum chloride disturbances are commonly observed in critically ill dogs with diverse disease etiologies (51, 54) and hyperchloremia is a common abnormality associated with metabolic acidosis in dogs. Because there was no evidence of dehydration and metabolic acidosis in patients enrolled in this study, interference by opioids was suspected, this is aligned with the findings of by Hopper and Epstein (55). However, unlike Hopper and Epstein (55), in our study we observed hypernatremia. Blood sodium values tend to rise for all opioids at T1, but by a greater percentage when methadone is administered. At T2, there is an increase in its value only with buprenorphine. Tramadol, on the other hand, has the opposite effect, with a decrease in the value of sodium and chloride at T2 compared to T1 which, as far as sodium is concerned, is even lower than those observed at T0. A profound hyponatremia was reported by Lota et al. (56) following a tramadol overdose and this is a common electrolyte disturbance associated with prolonged hospitalization and increased mortality. This certainly does not correspond to our case since the decrease between T0 and T2 is only 0.31%. It must be remembered, however, that the administration of intravenous chloride and sodium is ubiquitous in hospitalized veterinary patients and the electrolyte concentration, routinely measured in veterinary hospital practice, may suffer interferences related to fluid therapy.

A post-operative decrease in ionized calcium (iCa) was demonstrated in healthy animals after various anesthetic protocols and surgeries (57). In our case iCa shows a significant increase over time, except in the group treated with tramadol where the initial value remains constant. This different behavior is probably due to the different action on the calcium channels of the opioids used in our study. It could be assumed that the dilution of blood samples with heparin could be a source of preanalytical error in blood gas, electrolyte, and lactate measurements, but the use of tubes with lithium heparin is recommended for ionized calcium (58).

In addition to changes in the gas analytic parameters, the opioids used in this study were well tolerated without undesirable adverse effects.

Our study has limitations, sample sizes are relatively small. Prospective studies with a higher number of enlisted animals could provide more reliable data. Also, given the pharmacokinetics of these opioid analgesic drugs, it would be advisable to evaluate blood glucose several times a day.

## Conclusion

Opioid analgesic agents remain the first choice for common treatment of severe pain in everyday veterinary hospital practice. In hospitalized dogs, the hematological parameters that show the greatest impact from the use of opioids glucose, lactate and electrolytes. It follows that monitoring of gas analytic parameters should be a priority for patients in hospital and that more attention should be paid to hospitalized patients with certain metabolic and endocrine diseases.

## Data Availability Statement

The original contributions presented in the study are included in the article/supplementary material, further inquiries can be directed to the corresponding author.

## Ethics Statement

The animal study was reviewed and approved by Ethics Committee for Animal Experimentation of University of Parma (PROT. N.14/CESA /2021). Written informed consent was obtained from the owners for the participation of their animals in this study.

## Author Contributions

FQ: conceptualization, supervision, funding acquisition, writing-original draft preparation, and writing-review and editing. FF, KS, and RA: data curation. FQ, FF, and KS: writing-review and editing. All authors have read and agreed to the published version of the manuscript.

## Conflict of Interest

The authors declare that the research was conducted in the absence of any commercial or financial relationships that could be construed as a potential conflict of interest.

## Publisher's Note

All claims expressed in this article are solely those of the authors and do not necessarily represent those of their affiliated organizations, or those of the publisher, the editors and the reviewers. Any product that may be evaluated in this article, or claim that may be made by its manufacturer, is not guaranteed or endorsed by the publisher.
